# Feeding Behavior of Mice under Different Food Allocation Regimens

**DOI:** 10.1155/2019/1581304

**Published:** 2019-12-02

**Authors:** Hiroshi Ueno, Shunsuke Suemitsu, Shinji Murakami, Naoya Kitamura, Kenta Wani, Yu Takahashi, Yosuke Matsumoto, Motoi Okamoto, Takeshi Ishihara

**Affiliations:** ^1^Department of Medical Technology, Kawasaki University of Medical Welfare, Okayama 701-0193, Japan; ^2^Department of Psychiatry, Kawasaki Medical School, Okayama 701-0192, Japan; ^3^Department of Neuropsychiatry, Graduate School of Medicine, Dentistry and Pharmaceutical Sciences, Okayama University, Okayama 700-8558, Japan; ^4^Department of Medical Technology, Graduate School of Health Sciences, Okayama University, Okayama 700-8558, Japan

## Abstract

Social interaction, a basic survival strategy for many animal species, helps maintain a social environment that has limited conflict. Social dominance has a dramatic effect on motivation. Recent evidence suggests that some primate and nonprimate species display aversive behavior toward food allocation regimens that differ from their peers. Thus, we examined the behaviors displayed by mice under different food allocation regimens. We analyzed changes in food intake using several parameters. In the same food condition, the mice received the same food; in the quality different condition, the mice received different foods; in the quantity different condition, one mouse did not receive food; and in the no food condition, none of the mice received food. To test differences based on food quality, one mouse received normal solid food as a less preferred reward, and the other received chocolate chips as a high-level reward. No behavioral change was observed in comparison to the same food condition. To test differences based on food quantity, one mouse received chocolate chips while the other received nothing. Mice who received nothing spent more time on the other side of the reward throughout the experiment. Interestingly, highly rewarded mice required more time to consume the chocolate chips. Thus, under different food allocation regimens, mice changed their behavior by being more hesitant. Moreover, mice alter food intake behavior according to the social environment. The findings help elucidate potential evolutionary aspects that help maintain social cohesion while providing insights into potential mechanisms underlying socially anxious behavior.

## 1. Introduction

Social interaction is a basic survival strategy for many animal species. Social situations strongly impact behavior and motivation. Such situations can either stimulate behavior (social facilitation effects) or slow it down [1–3]. The present study examined how social contexts are related to feeding behavior. Specifically, we examined different food allocation regimens within a social context. This particular effect is sometimes referred to as “inequity aversion” in economics and other behavioral sciences [4, 5].

Mice are extremely social species and develop within hierarchically structured and well-organized social groups [6]. Therefore, we hypothesized that mice would change their food intake behavior under different food allocations. There is partial support for this idea whereby prosocial behavior, empathy, and inequity aversion could exist in rodents [7–9]. These capabilities are important for animals participating in group activities although they have historically been considered as chiefly present in humans and primates. Acting for others without expecting any external compensation is referred to as prosocial behavior [10]. Until recently, humans and primates have been considered the only beings with the requisite higher order cognitive capacities for prosocial behavior [11–13]. However, in recent years, it has been reported that prosocial rescue behaviors are performed by rodents [9, 14, 15]. Here, rescue behavior is likely based on empathic capacity [14, 16, 17]. Empathy has also been historically considered a high-level cognitive process only present in humans and primates. Yet, empathy has been observed in nonprimate species [18–20], birds [21, 22], and rodents [8, 23, 24]. Thus, it is becoming evident that animals other than humans also display empathy-like behaviors. In order to demonstrate empathy-like behavior, it is necessary to understand others' behaviors and emotions [25]. To this end, rodents have the potential to visually perceive others' behaviors [8].

Social facilitation in animals refers to how the presence and/or behavior of one animal increases the likelihood that other animals will engage in, or increase the intensity, of a specific behavior [1, 23]. Social facilitation occurs in a wide variety of species for a variety of situations such as eating, cleaning, teaching, sexual behavior, coalitions, and group behavior [26]. Given that eating is a very simple behavior, social facilitation is expected to result in an individual consuming more food if eating in the presence of other allogeneic individuals. The general phenomenon of social zfeeding facilitation has been demonstrated in a wide variety of species, including chickens, fish, rats, gerbils, puppies, and primates [27–32]. However, whether mice show such social facilitation under different food allocations has not been determined.

Within the human society, the failure to recognize social deficits and emotional states from a partner interferes with communication. Indeed, sensitivity to the emotional state of conspecifics is tremendously important for social animals. Impairments in higher order cognitive functions are characteristic of various neuropsychiatric disorders [26–28]. Examining the presence of higher cognitive functions and their underlying mechanisms in mice is important for furthering our understanding of human neuropsychiatric disorders. Indeed, it is possible to develop new therapeutic targets for these diseases through such work.

Therefore, the present study investigated whether mice change their behavior in response to different food allocation situations. We placed two mice on either side of a transparent wall and provided various foods. In the same food condition, the mice received the same food; in the quality different condition, the mice received different foods; in the quantity different condition, one mouse did not receive food; and in the no food condition, neither mouse received food. The variables analyzed were the latency to eat, total time taken to eat, distance traveled, and staying time.

## 2. Results

In various conditions of the feeding behavioral test, both target mice and cage-mate mice included for social facilitation were provided with 6 chocolate chips (Table 1). The parameters of latency to start eating and the distance traveled measured in this test were used.

### 2.1. Behavior in response to the Quality Different Condition

**Figure 1:**
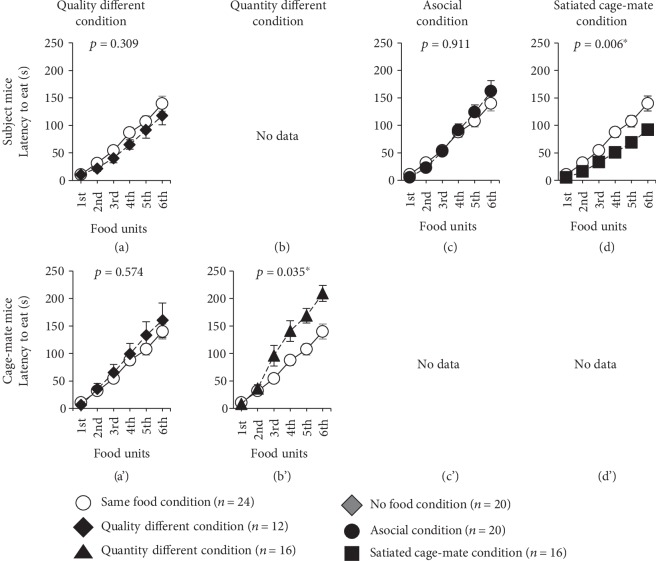
Comparison of the latency to begin eating 6 food units. Comparison of the latency to start eating the 6 food units in various conditions of the feeding behavioral test (same food condition, *n* = 24; quality different condition, *n* = 12; quantity different condition, *n* = 16; no food condition, *n* = 20; asocial condition, *n* = 20; stuffed cage-mate condition, *n* = 16). The upper row shows the subject mice ((a)–(d)), and the lower row shows the cage-mate mice ((a′)–(d′)). Data are presented as means ± SEM. Statistical significance is represented by asterisks: ^∗^*p* < 0.05. The *p* values were calculated using a two-way repeated measures ANOVA.

**Figure 2:**
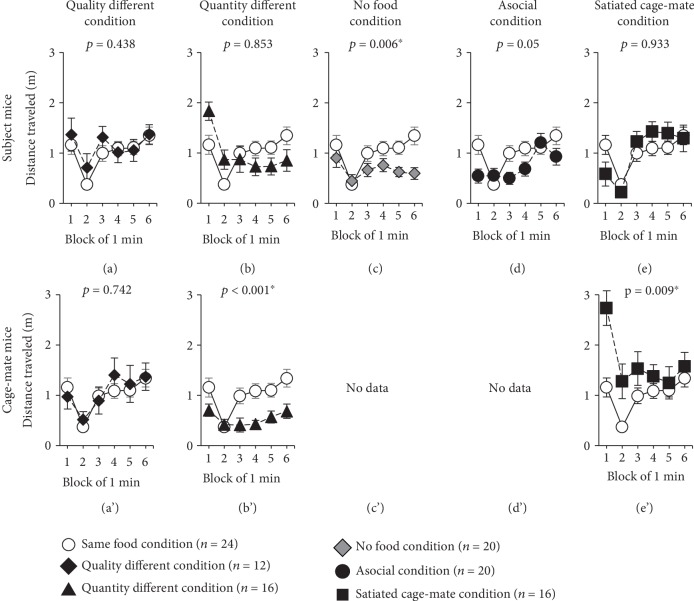
Comparison of the distance traveled per 1-minute period. Comparison of the distance traveled in each 1-minute period in various conditions of the feeding behavioral test (same food condition, *n* = 24; quality different condition, *n* = 12; quantity different condition, *n* = 16; no food condition, *n* = 20; asocial condition, *n* = 20; stuffed cage-mate condition, *n* = 16). The upper row shows the subject mice ((a)–(e)), and the lower row shows the cage-mate mice ((a′)–(e′)). Data are presented as means ± SEM. Statistical significance is represented by asterisks: ^∗^*p* < 0.05. The *p* values were calculated by a two-way repeated measures ANOVA.

**Figure 3:**
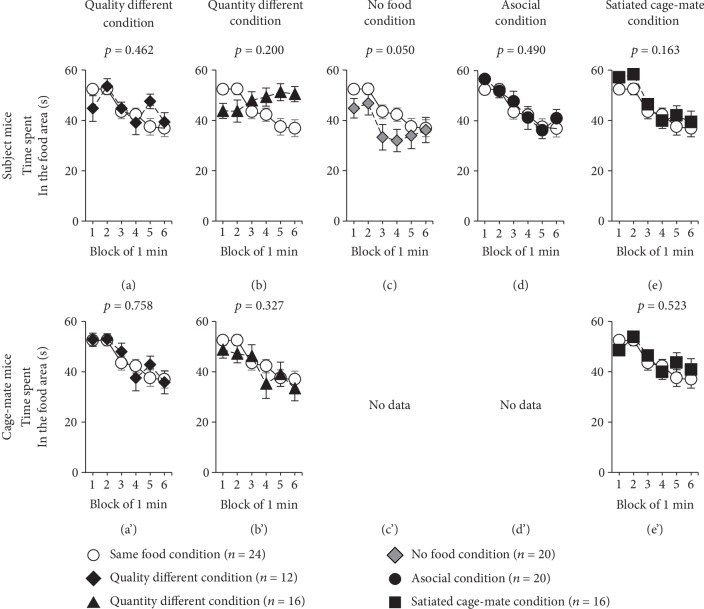
Comparison of the time spent per 1-minute in the food area. Comparison of the time spent in the food area in each 1-minute period in various conditions of the feeding behavioral test (same food condition, *n* = 24; quality different condition, *n* = 12; quantity different condition, *n* = 16; no food condition, *n* = 20; asocial condition, *n* = 20; stuffed cage-mate condition, *n* = 16). The upper row shows the subject mice ((a)–(e)), and the lower row shows the cage-mate mice ((a′)–(e′)). Data are presented as means ± SEM. Statistical significance is represented by asterisks: ^∗^*p* < 0.05. The *p* values were calculated by a two-way repeated measures ANOVA.

Subject mice were provided with solid foods, while chocolate chips were provided to the cage-mate mice (Table 1, [Supplementary-material supplementary-material-1] Video). Among subject mice, there was no significant difference in latency to eat (Figure 1(a), *F*_1,31_ = 1.069, *p* = 0.309), distance traveled in each 1-minute period (Figure 2(a), *F*_1,34_ = 0.616, *p* = 0.438), and time spent in the food area (Figure 3(a), *F*_1,34_ = 0.553, *p* = 0.462) as compared to the same food condition test.

Among cage-mate mice, there was no significant difference in the latency to eat (Figure 1(a)′, *F*_1,32_ = 0.323, *p* = 0.574), distance traveled in each 1-minute period (Figure 2(a)′, *F*_1,34_ = 0.110, *p* = 0.742), and time spent in the food area (Figure 3(a)′, *F*_1,34_ = 0.096, *p* = 0.758) as compared to the same food condition test.

### 2.2. Behavior in response to the Quantity Different Condition

Here, we provided nothing to the subject mice and chocolate chips to the cage-mate mice (Table 1, [Supplementary-material supplementary-material-1] Video). Among the subject mice, there was no significant difference in the distance traveled in each 1-minute period (Figure 2(b), *F*_1,38_ = 0.035, *p* = 0.853) and time spent in the food area (Figure 3(b), *F*_1,38_ = 1.700, *p* = 0.200) as compared to the same food condition test. Compared to the no food condition test, there was no significant difference in distance traveled in each 1-minute period (no figure, *F*_1,34_ = 3.717, *p* = 0.062). Regarding the time spent in the food area, subject mice spent a longer time in the food area as compared to the no food condition test (no figure, *F*_1,34_ = 6.203, *p* = 0.018).

Among the cage-mate mice, the latency to eat was significantly extended in the quantity different condition as compared to the same food condition test (Figure 1(b)′, *F*_1,34_ = 4.847, *p* = 0.035). The cage-mate mice traveled a significantly shorter distance in each 1-minute period during the 6-minute test period than they did in the same food condition test (Figure 2(b)′, *F*_1,38_ = 16.975, *p* < 0.001). There was no significant difference in the time spent in the food area (Figure 3(b)′, *F*_1,38_ = 0.987, *p* = 0.327) as compared to the same food condition test.

### 2.3. Behavior in response to the No Food Condition

Here, we provided nothing to both the subject mice and cage-mate mice (Table 1, [Supplementary-material supplementary-material-1] Video). Among the subject mice, the distance traveled in each 1-minute period significantly decreased in the no food condition as compared to the same food condition test (Figure 2(c), *F*_1,42_ = 8.222, *p* = 0.006). Additionally, there was no significant difference in the time spent in the food area (Figure 3(c), *F*_1,42_ = 4.081, *p* = 0.050) as compared to the same food condition test.

### 2.4. Behavior in response to the Asocial Condition

Here, chocolate chips were provided to the subject mice without any cage-mate mice present (Table 1). Among the subject mice, there was no significant difference in latency to eat (Figure 1(c), *F*_1,36_ = 0.013, *p* = 0.911), distance traveled in each 1-minute period (Figure 2(d), *F*_1,42_ = 4.071, *p* = 0.050), and time spent in the food area (Figure 3(d), *F*_1,42_ = 0.485, *p* = 0.490) as compared to the same food condition test.

### 2.5. Behavior in response to the Satiated Cage-Mate Condition

In this test, to determine subject mouse responses when a satiated cage-mate mouse is placed in the opposite lane, the subject mice received 6 chocolate chips (Table 1). Among the subject mice, the latency to eat was significantly reduced in the satiated cage-mate condition as compared to the same food condition test (Figure 1(d), *F*_1,37_ = 8.571, *p* = 0.006). There was no significant difference in the distance traveled in each 1-minute period (Figure 2(e), *F*_1,38_ = 0.007, *p* = 0.933) and time spent in the food area (Figure 3(e), *F*_1,38_ = 2.027, *p* = 0.163) as compared to the same food condition test.

The cage-mate mice traveled a significantly longer distance in each 1-minute period during the 6-minute test period than they did in the same food condition test (Figure 2(e)′, *F*_1,38_ = 7.685, *p* = 0.009). No significant difference was found in the time spent in the food area (Figure 3(e)′, *F*_1,38_ = 0.416, *p* = 0.523) as compared to the same food condition test. Compared to the no food condition test, the distance traveled in each 1-minute period was significantly extended in this test (no figure, *F*_1,34_ = 17.006, *p* < 0.001). In terms of the time spent in the food area, the cage-mate mice spent a longer time in this test than in the no food condition test (no figure, *F*_1,34_ = 4.961, *p* = 0.033).

### 2.6. Behavior in response to the Chocolate Chip Beyond the Wall Condition

**Figure 4:**
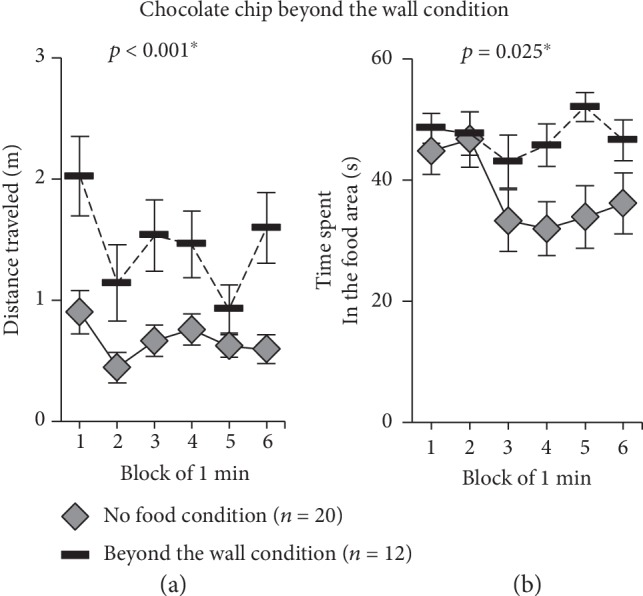
Feeding behavioral test of chocolate chip beyond the wall condition. Comparison of the distance traveled in each 1-minute period (a) (no food condition, *n* = 20; beyond the wall condition, *n* = 12). Comparison of the time spent in the food area in each 1-minute period (b). Data are presented as means ± SEM (a, b). Statistical significance is represented by asterisks: ^∗^*p* < 0.05. The *p* values were calculated by a two-way repeated measures ANOVA (a, b).

Subject mice traveled a significantly longer distance in each 1-minute period during the 6-minute test period than they did in the no food condition test (Figure 4(a), *F*_1,30_ = 16.870, *p* < 0.001, [Supplementary-material supplementary-material-1] Video). Time spent in the food area (Figure 4(b), *F*_1,30_ = 5.551, *p* = 0.025) was also significantly longer as compared to the no food condition test.

## 3. Discussion

This study was one of the first to investigate feeding behavior of mice under different food allocation regimens. Results revealed that mice exhibited different responses under different food conditions than under the same food conditions. The findings also indicate that mice are sensitive to different food allocation regimens embedded within social contexts. Furthermore, the mice were reluctant to earn rewards under profitable food conditions that varied allocations between peers. The findings suggest that mice have the ability to recognize and compare the context and circumstances of others and alter their behavior accordingly.

Changes in behavior under the various conditions observed in this study suggest that mice visually perceive and compare an opponent's behavior within a social situation. Past research indicates that mice show interest in cage-mates who display abnormal behaviors [24, 33]. In the present study, we posit that mice recognized the state of their peers across the transparent wall and altered behaviors as a result.

Under the quantity different conditions, mice who did not receive food appeared to be obsessed with locations where compensation was expected and traveled a further distance within the box. Even in comparison with the same food condition, where nothing was given to either mouse, the mouse that did not receive food was pointedly preoccupied with the spot where the rewards were expected. The behavior of these mice may possibly indicate recognition that the other party (on the other side of the transparent wall) is also not reaping any reward. Mice who did not receive food, as shown in the supplementary video, frequently touched the transparent wall and showed approach behaviors across the wall. Interestingly, dogs exhibit indicators of dislike when compared to nearby others and attempt to extract a reward [34]. Additionally, research has shown that Cebus paella show inequity-aversion behaviors when only receiving a small reward in conditions where a large reward should usually be obtained [35]. However, it is suggested that this reaction is not the same as inequity aversion but could rather be considered a behavior indicative of dissatisfaction [36, 37]. In the present study, we conducted experiments in the order shown in Table 1. Based on this design, we can exclude the possibility of dissatisfaction being displayed. The present results suggest that it is not pleasant for mice receiving no or small rewards to observe another mouse being satisfied with a large reward. This results in mice likely exhibiting inequity-aversion-like or envious behaviors. In the condition where mice did not receive any reward, and only the chocolate was placed behind a transparent wall (as compared to the no food condition), the mice tended to travel further distances and spent more time in front of the food. The mice showed dissatisfaction with the fact that they were not able to access the larger reward. In the quantity different condition, mice constantly lingered where the food would have been placed during the other conditions. The mice who had not received anything tended to also show a high interest in the foods that the other mice consumed. In a similar study using mice, a significant increase in body surface temperature due to quantitative inequality was reported [38], but this increase could also indicate physical exertion due to the increased travel distance present in this condition. However, considering the fact that mice observing other mice eating sometimes will experience hyperthermia [38], it is possible that the mice showed aversive behavior for two reasons: (1) no food could be obtained and/or (2) only the cage-mate consumed food. This seems to suggest that disgust was the negative emotional expression toward others expressed in these conditions. On the other hand, it is rare to find favorable positive emotional expressions toward others in such contexts [39]. Further research will be needed to clarify whether such happiness toward others is really experienced as unpleasant.

Within the quality different conditions, where the subject mice were given solid food and the cage-mate the chocolate, latency to eat, total time to eat, distance traveled, and time spent in the food area did not differ from the same food conditions. Even in experiments with dogs, behavioral differences under quality inequity conditions are not observed [34]. Conversely, Cebus paella recognize qualitative inequity and demonstrate aversive behaviors [40]. As such, it is unclear whether these behavioral differences are due to the experimental conditions, biological differences in the capacity to recognize qualitatively different conditions, or an interest in food. Further detailed assessments are necessary.

In the profitable different food condition, the subject mice received the chocolate chips, and the cage-mate mice received nothing. In this condition, the highly rewarded mice showed an increased latency to eat and total time to earn rewards, as well as a diminished total distance traveled. This is consistent with previous research observing that if subject mice only receive large rewards, they show increased body surface temperature despite the fact that they did not even attempt to earn the food [38]. This previous report raises the possibility that mice receiving large rewards recognize themselves as being happier than other mice, although there are other possible explanations. As indicated in the supplementary video, mice not receiving food across the wall frequently exhibited approach behaviors. The behavior of highly rewarded mice may have been influenced by the behavior of a mouse on the other side of the wall. It is possible that mice receiving large rewards experience psychological stress. Mice are animals that change their behavior by visually recognizing the state of conspecifics [8, 24, 41]. When receiving food outside the presence of others, time taken to eat and time staying in the food area did not change as compared with the same food condition. In the condition where satiated cage-mates were visible, total time to eat decreased. This indicates that behavior from the rewarded mouse changes depending on the status of the cage-mate. The reason that the highly rewarded mouse hesitated to earn foods could be a desire to avoid provoking jealousy from the cage-mate. It is unclear whether the decreased total distance traveled in such conditions was the byproduct of the extended eating time or avoiding cage-mates. Further research will be needed to clarify these possibilities. In a previous inequity-aversion behavioral study on monkeys and dogs, compensation was obtained in a few seconds, eating time was short, and an experimental method by which eating behavior could not be observed was used [34, 35]. Even in the present study, when one chocolate chip was provided as a large reward, no behavioral change was observed (data not shown). Therefore, it is important in the future to interpret food intake behavior by extending the time to earn the food as an indicator of an envious, evasive, or reserved-like behavior in a profit condition.

In the no food condition, we observed that both mice decreased the distance traveled and time spent in the food area as compared to the quantity different condition. The first two minutes of the test were considered a “long stay” in the food spot. There was no noticeable behavioral change, such as a fixation with the typical food spot, when there was no food for both mice. This result suggests that mice recognize that their status is equitable to their conspecific.

Mice are social animals and form hierarchically organized groups [42, 43]. This social stratification may have been linked to changes in food intake behavior within different food allocation regimens. The inequity-aversion responses that are disadvantageous are useful when selecting partners for sustaining a social life [40, 44, 45]. For social species, a negative response to inequality should help select future partners for promoting the establishment of a credible cooperative alliance [46]. Being sensitive to the value of an outcome may be an important part of maintaining a balanced social structure that helps ensure beneficial cooperation [47, 48]. Utilizing these animal models will help enable the study of psychological and biological bases of social motives. For instance, striatal nerve activity in the basal ganglia is sensitive to incentive contrast processes and preferences [49–52]. The medial prefrontal cortex and the amygdala are two other brain areas that are sensitive to social rewards [53–55].

One relevant known type of social anxiety disorder (SAD) is deipnophobia [56]. Patients with deipnophobia have a strong resistance to eating with others. The biological mechanism by which SAD (including deipnophobia) occurs is still unknown [57]. However, it is thought that deipnophobia is caused by a combination of external (trauma) and internal (genetic) factors [58]. As the causes of such diseases are unknown, effective drugs have not been developed, but the development of such therapies is expected in the future. Therefore, it is important to further investigate the neural mechanisms of this disease. The present study strongly indicates that mice are influenced by the status of a proximal cage-mate obtaining a reward. It is possible that social animals may have a mechanism for ingesting compensation in consideration of the gaze of conspecifics. Thus, results of the present study may contribute to clarifying the underlying mechanisms of SAD, including deipnophobia. Several animals avoid behaviors that are indicative of greed (e.g., appetite, sexual desire, sleep, and defecation) when observed by others. While these behaviors may emerge as a function of exposing one's vulnerability, the present study suggests that such animals may become stressed and provoke envy from observers. Avoiding stress and jealousy is vital to sustaining a social life. These avoidance behaviors (i.e., actions aimed at avoiding jealousy) are designed for self-protection. Thus, it is reasonable to posit that these abilities are commonly present in social animals.

## 4. Conclusion

We observed that mice alter food intake behaviors according to the social environment. Furthermore, it was revealed that mice exhibit hesitation-like behaviors in social feeding contexts. Social interaction supports the formation of large social groups, suggesting the possibility of these capacities being present in many animals. The present study provides a useful account to aid the elucidation of evolutionary aspects underlying motivation that maintains social cohesion.

## 5. Materials and Methods

### 5.1. Ethical Approval

This article does not contain any studies with human participants performed by any of the authors. All animal experiments were performed in accordance with the U.S. National Institutes of Health (NIH) Guide for the Care and Use of Laboratory Animals (NIH Publication No. 80-23, revised in 1996) and approved by the Committee for Animal Experiments at Kawasaki Medical School Advanced Research Center.

### 5.2. Animals

All animal experiments were performed in accordance with the U.S. National Institutes of Health (NIH) Guide for the Care and Use of Laboratory Animals (NIH Publication No. 80-23, revised in 1996) and approved by the Committee for Animal Experiments at Kawasaki Medical School Advanced Research Center (17-070). All possible efforts were made to ensure that we minimized the number of animals used and their suffering. Animals were purchased from Charles River Laboratories (Kanagawa, Japan) and housed in cages (five animals per cage) with food and water provided *ad libitum* under a 12 h light/dark cycle at 23°C–26°C. We used C57BL/6N male mice aged 15 weeks. All behavioral tests were conducted in behavioral testing rooms between 08:00 and 18:00 h during the light phase of the circadian cycle. After testing, all equipment was cleaned with 70% ethanol and super hypochlorous water to prevent potential bias based on olfactory cues.

### 5.3. Testing Apparatus of Feeding Behavioral Tests

**Figure 5:**
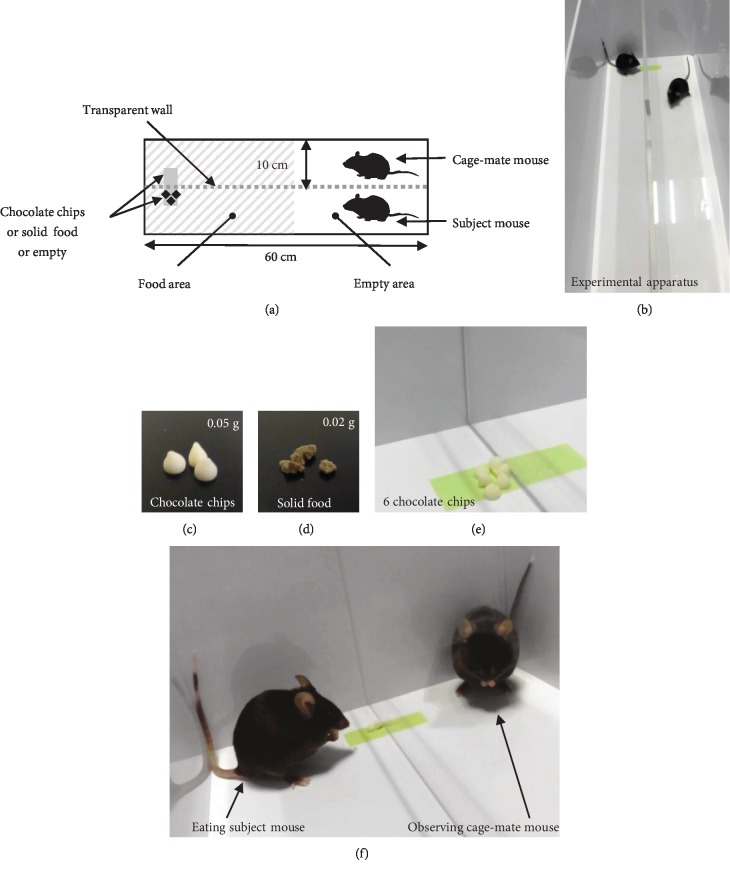
Testing apparatus of the feeding behavioral test. (a) Schematic diagram of the feeding behavioral test. Two mice were placed at the end of the lane simultaneously. The rewards were placed on the opposite side of the lanes. (b) Sample picture during the feeding behavioral test. The test mouse freely moved in the lane. (c) A sample image of the chocolate chips as a high-appeal reward, 0.05 g per unit. (d) A sample image of the ordinary crushed solid food as a low-appeal reward, 0.02 g per unit. (e) A sample picture of the 6 chocolate chips in the testing apparatus. (f) A sample picture of the feeding behavioral test. Subject mice and cage-mate mice were separated by transparent acrylic walls.

The testing apparatus consisted of a rectangular (20 × 60 × 40 cm) box (Figures 5(a) and 5(b)). Transparent acrylic walls (40 × 60 cm) were placed in the center of the rectangular box (Figures 5(a) and 5(b)). We prepared chocolate chips, which were high-appeal rewards and ordinary crushed solid food, which were low-appeal rewards (Figures 5(c) and 5(d)). The high-reward unit was 0.05 g per one chocolate chip. To synchronize the time to consume one reward unit, we used 0.02 g crushed solid food per low-reward unit. The rewards provided were consistently placed in one specific spot (Figures 5(a) and 5(e)). In all conditions, both rewards were visible to all subject mice (Figure 5(f)).

### 5.4. Testing Procedures of Feeding Behavioral Tests

The experiments were conducted with one mouse in each lane of the apparatus, and the two mice were put at the end of the lane simultaneously (Figure 5(a)). Each mouse was allowed to move freely in the lane for 6 minutes. The apparatuses were cleaned after each phase of the test. The rewards were placed on the opposite side of the lanes. We examined the response of each mouse to rewards in various conditions (Table 1). Behavioral tests were performed according to the test order described in Table 1. On the day before the start of the experiment, each mouse was placed in the box for 15 minutes and allowed free exploration to habituate. We performed experiments of one condition once a day for 7 days.

(1) In the same food condition test, to determine their response to the same reward, both subject mice and cage-mate mice received 6 chocolate chips. (2) In the test for the ability to detect differences in quality, subject mice received 6 solid foods, and cage-mate mice received 6 chocolate chips. (3) In the test for the ability to detect differences in quantity, subject mice received no foods, and cage-mate mice received 6 chocolate chips. (4) In the no food condition test, both subject mice and cage-mate mice received no rewards. (5) In the asocial condition test, subject mice received 6 chocolate chips and cage-mate mice were not used. (6) In the satiated cage-mate condition test, subject mice received 6 chocolate chips and a fully satiated cage-mate mouse was placed in the opposite lane. To satiate the cage-mate mice, they were allowed to eat chocolate chips freely for 15 minutes. (7) In the beyond the wall condition test, subject mice were not rewarded, and 6 chocolate chips were placed behind a transparent wall.

The food area refers to the side with rewards; the empty area refers to the side with no rewards (Figure 5(a)). The latency to start eating each of the 6 food pieces during the 6-minute sessions was measured. We also analyzed the distance traveled (m) and time spent in each area (s). Data were recorded on video and analyzed using video tracking software (ANY-MAZE, Stoelting Co., Wood Dale, IL).

### 5.5. Statistical Analyses

Statistical analyses were conducted using SPSS version 25 (IBM Corp., Tokyo, Japan). The data were analyzed using repeated measures ANOVA. A *p* value < 0.05 was regarded as statistically significant. Data were expressed as means ± SEM.

## Figures and Tables

**Table 1:** 

Conditions		Subject mice		Cage-mate mice	
Social or asocial	Test conditions	Reward	No.	Reward	No.
Social conditions	Same food condition	Chocolate chips	6	Chocolate chips	6
	Quality different condition	Solid food	6	Chocolate chips	6
	Quantity different condition	Empty		Chocolate chips	6
	No food condition	Empty		Empty	
Asocial condition		Chocolate chips	6	No cage-mate	
Satiated cage-mate condition		Chocolate chips	6	Satiated cage-mate	
Beyond the wall condition		Empty		Chocolate chips only	

## Data Availability

All relevant data are within the manuscript.

## Supplementary Materials

Supplementary 1 Video 1: feeding behavioral test of the same food condition. This is a 4x speed movie.

Supplementary 2 Video 2: feeding behavioral test of the quality different condition. This is a 4x speed movie.

Supplementary 3 Video 3: feeding behavioral test of the quantity different condition. This is a 4x speed movie.

Supplementary 4 Video 4: feeding behavioral test of the no food condition. This is a 4x speed movie.

Supplementary 5 Video 5: feeding behavioral test of chocolate chip beyond the wall condition. This is a 4x speed movie.
